# Ghost Ileostomy with or without abdominal parietal split

**DOI:** 10.1186/1477-7819-9-92

**Published:** 2011-08-18

**Authors:** Michele Cerroni, Roberto Cirocchi, Umberto Morelli, Stefano Trastulli, Jacopo Desiderio, Mario Mezzacapo, Chiara Listorti, Luigi Esperti, Diego Milani, Nicola Avenia, Nino Gullà, Giuseppe Noya, Carlo Boselli

**Affiliations:** 1 Department of General Surgery, University of Perugia, St. Maria Hospital, Terni, Italy; 2 Department of General and Oncologic Surgery, University of Perugia, Perugia, Italy; 3 Academic Surgery Unit, Colorectal Surgery Department, Royal London Hospital, London, UK

**Keywords:** Rectal cancer, Surgery, Anastomotic leakage, Ghost ileostomy

## Abstract

**Background:**

In patients who undergo low anterior rectal resection, the fashioning of a covering stoma (CS) is still controversial. In fact, a covering stoma (ileostomy or colostomy) is worsened by major complications related to the procedure, longer recovery time, necessity of a re-intervention under general anesthesia for stoma closure and poorer quality of life. The advantage of Ghost Ileostomy (GI) is that an ileostomy can be performed only when there is clinical evidence of anastomotic leakage, without performing further interventions with related complications when anastomotic leak is absent and therefore the procedure is not necessary. Moreover, in case of anastomotic dehiscence and necessity of delayed stoma opening, mortality and morbidity in patients with GI are comparable with the ones that occur in patients which had a classic covering stoma. On the other hand, is simple to think about the possible economic saving: avoiding an admission for performing the closure of the ileostomy, with all the costs connected (OR, hospitalization, post-operative period, treatment of possible complications) represents a huge saving for the hospital management and also raise the quality of life of the patients.

**Methods:**

In this study we prospectively analyzed 20 patients who underwent anterior extra-peritoneal rectum resection for rectal carcinoma with TME and fashioning of GI realized with or without abdominal parietal split.

**Results:**

In the group of patients that received a GI without split laparotomy mortality was absent and in one case an anastomotic leak occurred. In the group of patients in which GI with split laparotomy was fashioned, one death occurred and there were one case of infection and one respiratory complication. Clinical follow-up was 12 months.

**Conclusions:**

The use of different techniques for fashioning a GI do not present significant differences when they are performed by expert surgeons, but further evidence is needed with more randomized trials, in order to have more data supporting the clinical observation.

## Background

The surgical treatment of lower rectal cancers has evolved from abdominoperineal resection to proctectomy with TME and colo-anal anastomosis. The main drawback of colo-anal anastomosis is the risk of leakage, which is reported to occur in 2.9%-20% of cases [1].

A covering stoma (CS) after low anterior rectal resection reduces the incidence of anastomotic leak and urgent re-intervention for complications related to colorectal anastomosis [2].

Even if a covering stoma is performed by the vaste majority of surgeons in order to protect the colorectal anastomosis, the decision of creating a CS is yet left to the personal experience of the surgeon which will analyze, during the operation, the safety of the anastomosis evaluating the blood supply and the eventual tension [3].

The presence of defunctioning stoma has more advantages in the subgroup of patients that are at high risk of anastomotic leak: patients with low anastomosis or that previously underwent radio-chemotherapy [4].

Nevertheless, the advantages of a CS are reduced by the stoma-related complications or by the necessity of a re-intervention for the closure of that stoma, with subsequent increase of costs and recovery time. The overall incidence of clinical leak is 8%, therefore CS is confectioned and opened in 92% of cases, in the vaste majority of them, if analized retrospectively, with minimal or no clinical usefulness [5].

Some surgeons have recently suggested the creation of a pre-stage ileostomy (Ghost Ileostomy - GI) [6]: an intestinal loop of terminal ileum is identified, maintaining a proper blood supply and freed by any tension on the vascular pedicle, and exteriorized passing through an opening in the mesenteric border (preserving the vascular arcade) either a vascular vessel loop or with a pediatric Robinson catheter (cutting it 0.5 cm from the connection side, which is discarded), which is exteriorized through an classic ostomy opening through the abdominal wall, tension-free, and then fixed to the skin with two stitches of non absorbable suture. The GI is covered with a non adherent dressing (like Gelonet^®^) and observed daily. In case of clinical evidence of anastomotic leak [7], the GI can be opened and transformed into a classical covering ileostomy. Otherwise, if there is no evidence of anastomotic leakage, the vessel loop or the pediatric Robinson catheter are cut, the small bowel loop is repositioned in the abdominal cavity and surgical wound is closed layer by layer, starting from the fascia. This could happening some situations also without using general anaesthesia, only with local anaestetics and mild sedation in an a adequately set-up and equipped pre-anaestetic room, being ready to enter the operating theatre if any complication arise at the moment of closing the GI.

Confectioning of GI can be performed with different techniques. The aim of our study is to evaluate the surgical techniques that are currently employed and analyze the eventual benefits and complications that each procedure carries.

## Materials and methods

We prospectively analyzed 20 patients who underwent anterior extra-peritoneal resection of the rectum for rectal carcinoma with TME and fashioning of GI realized with or without abdominal parietal split.

**Figure 1:**
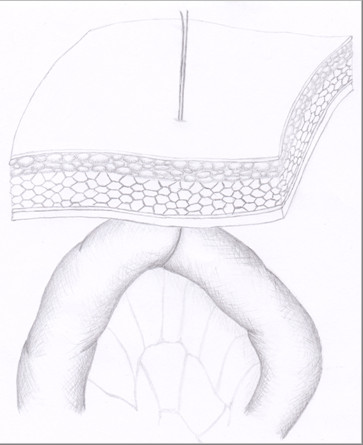
**Ghost ileostomy without parietal split**. The second-to-last ileal loop is intraoperatively marked with a Prolene^® ^stitch and the thread was then exteriorized with a Reverdin needle through the abdominal wall in the right iliac fossa.

**Figure 2:**
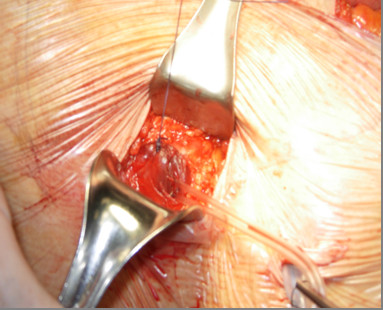
**Ghost ileostomy with parietal split**. A Mc Burney incision is made in the right iliac fossa.

**Figure 3:**
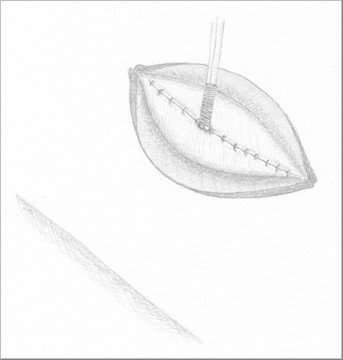
**Ghost ileostomy with parietal split**. The surgical incision is sutured in layers around the pediatric Robinson catheter.

**Figure 4:**
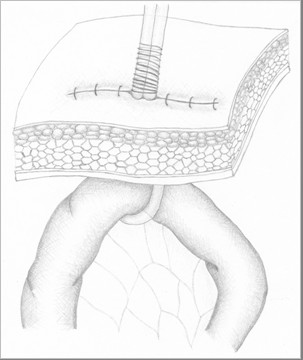
**Ghost ileostomy with parietal split**. The pediatric Robinson catheter is fixed to cutaneous surface.

**Figure 5:**
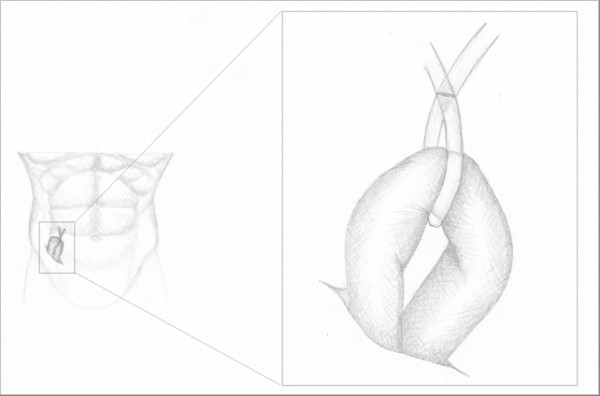
**In case of anastomotic leakage the intestinal loop is exteriorized through the abdominal laparotomy in right iliac fossa**.

The two groups of patients were homogeneous for age (54-86 years) and sex (12 males), and rectal adenocarcinoma was staged in both groups as T2-T3 localized at ≤ 10 cm from anal verge. In all the patient staged as T3 (N = 9) neoadjuvant radio-chemotherapy was performed. Laparotomical extra-peritoneal anterior resection (AR) of the rectum with TME was performed within 6 weeks after radio-chemotherapy. A GI was fashioned after AR and the realization of the colorectal anastomosis. In the group of patient where a GI without abdominal parietal split was realized (N = 10), the second-to-last ileal loop was intraoperatively marked with a Prolene^® ^stitch and the thread was then exteriorized with a Reverdin needle through the abdominal wall in the right iliac fossa. The intestinal loop was verified to be not under tension by the operating surgeon and then left just under the fascial layer ready to be eventually exteriorized. The Prolene^® ^thread was then stitched to the skin with non-absorbable suture stitches (Figure 1). In the group of patients in which a GI with abdominal parietal split group (N = 10) was fashioned a Mc Burney incision is made in the right iliac fossa (Figure 2). The next-to-last ileal loop is identified with a pediatric Robinson catheter, which is then exteriorized through the incision. The surgical incision is subsequently sutured in layers around the pediatric Robinson catheter (Figure 3). Intestinal loop is left "hanging" into the abdominal wall free from tension. The pediatric Robinson catheter is then fixed to cutaneous surface with not absorbable suture stitches (Figure 4). The pediatric Robinson catheter is removed in post-operative day 9-10 in case no anastomotic leak occurs, otherwise the suture stitches closing the incision in the right iliac fossa are removed, the intestinal loop with the pediatric Robinson catheter is exteriorized and the ileostomy is fashioned under local anesthesia (Figure 5).

## Results

In the group of patients that received a GI without split laparotomy, in one case an anastomotic leak occurred, for which surgical intervention under general anesthesia was required and a traditional loop ileostomy was fashioned. The leakage occurred in post-operative day 7 with findings of fecaloid material mixed with pus coming out from the peri-anastomotic drainage positioned during the surgical intervention. The patient was put under Total Parental Nutrition (TPN) and a full course of antibiotic therapy. Seven months later the patient underwent the closure of the ileostomy with good results. Mortality was absent.

In the group of patients in which GI with split laparotomy was fashioned, one case of infection of the surgical wound and one respiratory complication occurred (bilateral lower lobe consolidation with bronchopneumonia). No clinically detectable leakage occurred. One death occurred for myocardial infarction. Clinical follow-up was 12 months. One hernia occurred at the site of median laparotomic scar in the GI without split laparotomy group.

## Discussion

In Western Countries colorectal cancer is the third malignant tumor for incidence and mortality, after breast cancer in women and lung cancer in men. Colorectal cancer is rare after 40 years of age and is more frequent from 60 years of age; it reaches a peak around 80 years of age and men and women are equally affected. The number of tumors has increased whereas mortality has decreased, mostly because of more adequate information, early diagnosis and therapy improvements [8]. Gold standard in the treatment of rectal cancer is anterior rectal resection with TME, using the open technique or laparoscopy [9]; the latest studies underlined the importance of preserving the anatomy and the function also for low rectal malignancies, with new approaches to the question AR vs. APR [10].

Colorectal anastomosis leak is the most frequent complication of surgery for the treatment of rectal cancer (11% over 24,854 patients in a recent systematic revision) [11]. The incidence of leaks mainly depends on height of the anastomosis (< or = 6 cm) [12], preoperative radio-chemotherapy (10.9%) and surgical experience (2.9% in expert surgeons) [13-16].

The International Study Group of Rectal Cancer defines anastomotic leak as a "defect of the intestinal wall integrity at the colorectal or colo-anal anastomotic site (including suture and staple lines of neorectal reservoirs) leading to a communication between the intra- and extraluminal compartments [14-16]. The International Study Group of Rectal Cancer has also defined the grade of anastomotic leak in relation to the treatment [17]; the risk of re-intervention for permanent stoma after anastomotic leak is very high (25%) [18]. In this study the authors diagnosed the leakage when two or more of the following clinical parameters (routinely analized in all the patients which underwent colorectal surgery) were found in the postoperative course: raised WBC, raised CRP (plasmatic C-reactive protein), abdominal pain, prolonged ileus, fecaloid material or pus drained by the peri-anastomotic drain, raised temperature, generalized signs of sepsis. In our analysis the number of patients treated with ghost ileostomy with or without split laparotomy is small. This naturally represents a limit of the study and prevents the evaluation of all the advantages of the two techniques from a statistical point of view, but considering this as a small explorative pilot study some observations could be done:

- Exteriorization of GI under local anesthesia in ghost ileostomy with split laparotomy (removal of suture stitches previously used for closure of the layers, exteriorization of intestinal loop using the pediatric Robinson catheter as guide and fashioning the ileostomy) vs exteriorization of GI under general anesthesia in GI fashioned without split laparotomy (surgical incision in the right iliac fossa, exteriorization of intestinal loop tractioning the Prolene^® ^thread and fashioning the ileostomy). The two techniques does not present particular technical pitfalls, but the need of general anaesthesia in the second case should be considered especially in patients with elevated ASA score.

- Risk of wound suppuration and hernias only in patients with ghost ileostomy with split laparotomy.

Even if our study is not randomized, observational evidence shows that the GI with or without split laparotomy is a feasible alternative to the classical loop ileostomy and potentially has the same advantages and disadvantages when it is performed by trained surgeons with a minimal learning curve.

## Conclusion

In patients who undergo low anterior rectal resection, confectioning of CS is still controversial. In fact, covering stoma (ileostomy or colostomy) is characterized by major complications related to the procedure, longer recovery time, necessity of a re-intervention under general anesthesia for stoma closure and worse quality of life [2,14].

The advantage of GI is that ileostomy can be performed only when there is clinical evidence of anastomotic leakage [19], without performing further interventions with related complications when anastomotic leak is absent and therefore the procedure is not necessary. Moreover, in case of anastomotic dehiscence and necessity of delayed stoma opening, mortality and morbidity in patients with GI are comparable with the ones that occur in patients which had a classic covering stoma. On the other hand, is simple to think about the possible economic saving: avoiding an admission for performing the closure of the ileostomy, with all the costs connected (OR, hospitalization, post-operative period, treatment of possible complicances) represents a huge saving for the hospital management and also raise the quality of life of the patients The use of different techniques for fashioning a GI do not present significant differences when they are performed by expert surgeons, but further evidence is needed with more randomized trials, in order to have more data supporting the clinical observation.

## Competing interests

The Authors state that none of the authors involved in the manuscript preparation has any conflicts of interest towards the manuscript itself, neither financial nor moral conflicts. Besides none of the authors received support in the form of grants, equipment, and/or pharmaceutical items.

## Authors' contributions

All authors contributed equally to this work, read and approved the final manuscript.
